# The Review of Insulin Pens—Past, Present, and Look to the Future

**DOI:** 10.3389/fendo.2022.827484

**Published:** 2022-03-08

**Authors:** Małgorzata Masierek, Katarzyna Nabrdalik, Oliwia Janota, Hanna Kwiendacz, Maksymilian Macherski, Janusz Gumprecht

**Affiliations:** ^1^ Department of Internal Medicine, Diabetology and Nephrology, Faculty of Medical Sciences in Zabrze, Medical University of Silesia, Katowice, Poland; ^2^ Students’ Scientific Association by the Department of Internal Medicine, Diabetology and Nephrology, Faculty of Medical Sciences in Zabrze, Medical University of Silesia, Katowice, Poland

**Keywords:** insulin, pen, diabetes mellitus, prefilled pen, smart pen

## Abstract

Currently, there are about 150–200 million diabetic patients treated with insulin globally. The year 2021 is special because the 100th anniversary of the insulin discovery is being celebrated. It is a good occasion to sum up the insulin pen technology invention and improvement which are nowadays the leading mode of an insulin delivery. Even though so many years have passed, insulin is still administered subcutaneously, that is why devices to deliver it are of great importance. Insulin pens have evolved only through the last decades (the reusable, durable pens, and the disposable, prefilled pens) and modern smart insulin pens have been developed in the last few years, and both types of the devices compared to traditional syringes and vials are more convenient, discrete in use, have better dosing accuracy, and improve adherence. In this review, we will focus on the history of insulin pens and their improvement over the previous decades.

## Introduction

The International Diabetes Federation (IDF) estimates that over 537 million people all over the world are currently struggling with diabetes mellitus (DM) (1) and there are about 150–200 million of them treated with insulin (2). The history of insulin dates back to the last century, when in 1921 Frederick Banting and Charles Best with the support of John Macleod and James Collip discovered insulin and thereby revolutionized the treatment of DM (3–5). The first injection of insulin on January 11, 1922, to a 14-year-old boy with the use of reusable glass-bodied syringes (6) started an entirely new era of diabetes management (4, 5) and led to the improvement of insulin delivery methods (3, 5). Even though insulin has been used for 100 years already, its administration remains subcutaneous where insulin pens which evolved only through the last four decades are the leading method of its delivery (about 60% of patients treated with insulin use insulin pens all over the world) (7–9). Insulin pen utility is not the same in different regions of the world. According to a report from the year 2008, insulin pens were used by only 15% of patients in the US, compared with 80%–90% in Europe, and it was suspected that it could be due to limited education regarding the benefits of insulin pens but also their higher price (10). The situation has changed in the next years where data from the year 2011 indicate that the number of patients initiating vial/syringe in the US decreased from 2005 to 2011 to approximately 30% while patients initiating pens increased to approximately 60% (11). According to a IQVIA^®^ report for the period from June 2020 till June 2021 prepared for the purpose of this manuscript [data not published (12)], the usage of pens in US rose to 59% where in Europe it is comparably high and assessed to be 93.6%. Insulin pens have numerous advantages over traditional vial and syringe injections, among others easy use especially for patients with vision problems or manual dexterity, accuracy of delivering small doses of insulin, and discretion of use (13). It is worth noting that aspects of insulin administration may also contribute to the treatment outcomes even though the type of insulin and its efficacy and safety are the primary factors to consider. It is important to underline that each insulin-producing company has its own insulin pen dedicated to use with the produced insulin. It was proved in some studies that patients who use insulin pens are more adherent to the treatment regimen and have less hypoglycemic events compared to insulin vial users (14–18). Also, numerous studies report that patients’ preference for insulin pens exceeds that for vials or syringes (19–21) and portability of insulin pens improves patients’ convenience (22). However, it is important to note that the superiority of insulin pens in achieving and maintaining glycemic control has been questioned, and this question has not been resolved up to day (23). American and European guidelines underline the necessity of undertaking patient preference when selecting diabetes treatment especially when treatment is accoutered with pain due to injection (24). That is why recently a study assessing the patient perspective of injectable treatment among patients with type 2 diabetes (T2DM) has been performed and showed that there are some features of the injection device that patients choose more often which may help in future improvement of insulin pens (25). Development of insulin pens is parallel to the development of newer insulin formulation where insulin pen must adapt to changes to dosing and timing requirements like it is in case of modern ultra-long-acting insulin analogue icodec, administered once weekly, which is under development (26). This year, the discovery of insulin turns 100 years, and this provides an opportunity to reflect on its administration methods over the past years.

**Figure 1:**
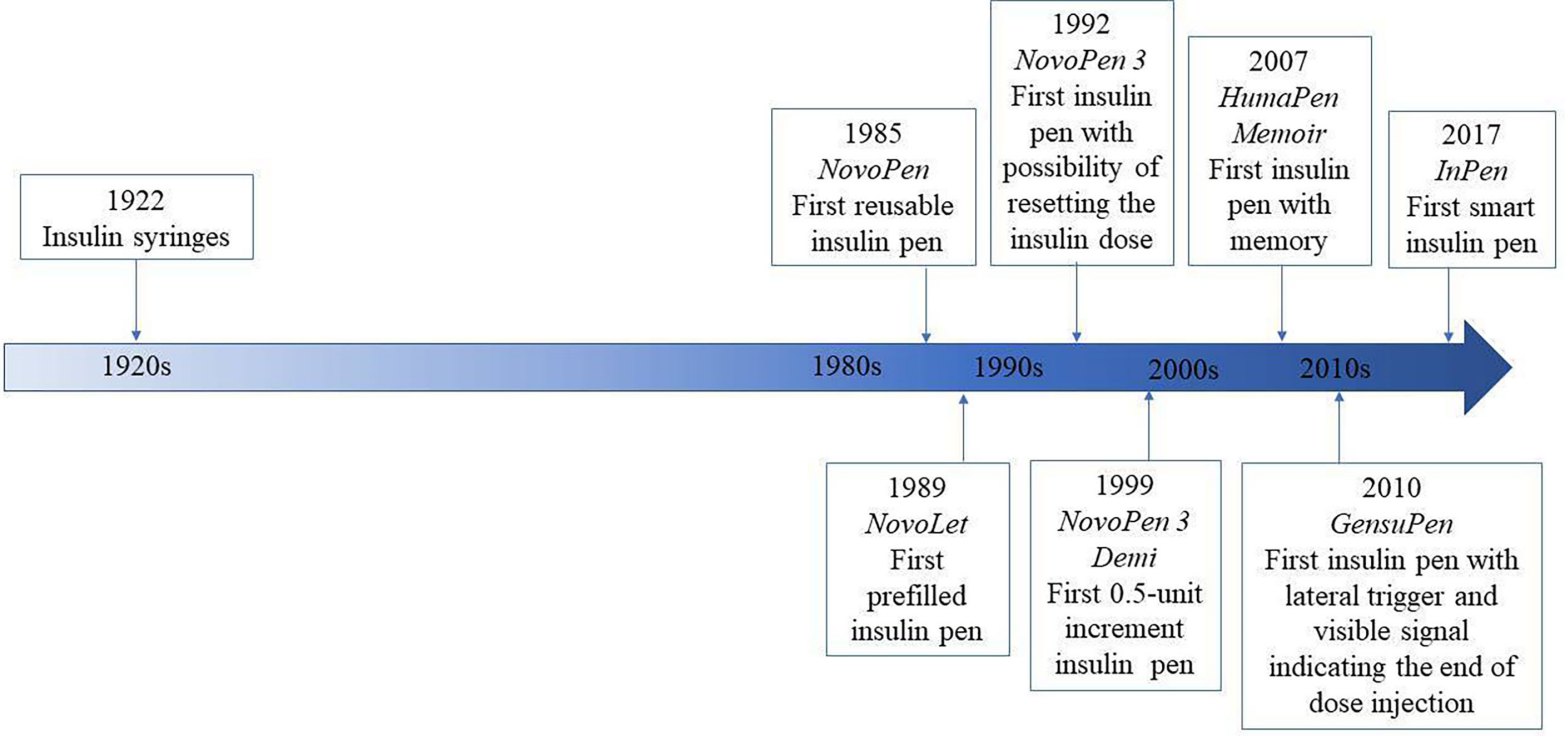
Timeline of insulin pen history.

In this review, we will focus on the history of insulin pens and their improvement over the previous decades, starting from the first-generation insulin pens throughout modern smart insulin pens (**Figure 1**). It must be noticed that clinical trials in relation to the newest smart insulin pens and insulin pen caps are very limited to date, that is why information related to this new technology comes also from manufacturer websites and commercial data resources.

## First-Generation Insulin Pens

The first insulin injections were made with large and heavy glass or metal syringes and reusable needles (4, 7, 24–26). Syringe was the only possible way of delivering insulin in clinical practice for the next several decades (4, 7, 27). This method of administration had several and serious disadvantages including poor dose accuracy, lack of social acceptance, and fear of injections (7, 27, 28). These inconveniences of the vial and syringe led to the manufacture of insulin pens. Majority of insulin pens are proprietary devices and are developed to work with specific insulin from the same manufacturer (29). Insulin pens are classified into two categories: being reusable (durable) or prefilled (disposable). The reusable insulin pen is loaded by the patient with replaceable insulin cartridges, and the prefilled insulin pen has the insulin reservoir cartridge already installed and the pen is discarded when the cartridge is empty. Both types of insulin pens can contain a maximum of 3 ml of insulin (30) and can deliver insulin in 0.5-, 1-, or 2-unit (U) increments up to 160 U with the use of a needle which has to be attached to the insulin pen.

## Reusable Insulin Pens

In 1985, Novo Nordisk has launched the first reusable insulin pen injector called NovoPen^®^ to overcome barriers of the vial and syringe (31) and started a series of NovoPen^®^ insulin injectors. The new device was a combination of the syringe and insulin vial in one mechanism, resembling a fountain pen (31). NovoPen^®^ contained a disposable, replaceable 1.5-ml insulin cartridge connected with a single-use needle and one-unit incremental dosing (29, 30) which was ready to use whenever needed. This allowed patients to administer multiple, preprandial injections discreetly, and their daily schedule became more flexible (32–34). First studies related to insulin pen comprised only several patients in 1995 (31), but as the development of the devices has grown up, also the number of patients studied increased to several hundreds per study in 2002 (35) and up to several thousands in 2020 (27, 36). Initially, insulin cartridges dedicated to insulin pen contained short-acting insulin for numerous injections before meals and basal insulin was injected with conventional syringes (37). Soon after, in 1988 a new insulin pen NovoPen^®^ 2 was presented to administer NPH and premixed insulins (38–40). Analogically as with short-acting insulins, majority of patients using the device to administer basal or mixed insulin preferred to continue the therapy with pens (38–40). In 1992, NovoPen^®^ 3 was launched which had a maximum dose that could be administered at one time which increased to 70 U (from 36 U with NovoPen^®^ 2) and the dialed doses could be reset without insulin waste. Soon after, in 1996 NovoPen^®^ 1.5 was released which had a smaller insulin cartridge and was shorter in length, followed by NovoPen^®^ 3 Demi to administer 0.5 U dose increments in 1999 and NovoPen^®^ Junior in 2003 which was designed with vibrant colors and developed specifically for children with diabetes. In 2005, NovoPen^®^ 4 was introduced which required reduced force to perform an injection, which had dose increments of 1.0 U and a maximum dose of 60 U (41). Moreover, NovoPen^®^ 4 was reported as simpler to learn and easier to use for both insulin-naïve and currently using NovoPen^®^ 3 patients (42). Following the release of NovoPen^®^s, other manufacturers have also introduced reusable insulin pens, including the HumaPen^®^ range (Eli Lilly and Company, Indianapolis, IN, USA) and the OptiPen^®^ Pro, OptiClik^®^, and ClikSTAR^®^ pens (Sanofi, Bridgewater, NJ, USA) The inconvenience of the first insulin pens was no possibility of dialing backward without wasting insulin, but the thing changed with the introduction of NovoPen^®^ 3 and HumaPen Ergo^®^ (35, 41). This option translated to device acceptability in comparison with previous generations of insulin injectors and syringes (43). With time, the option of insulin-free dialing forward and backward became a prevailing way of setting the insulin doses. All mentioned insulin pens had the trigger placed at on the opposite site of the needle attach end, but there are also insulin pens with a side-mounted release button used for half-automatic insulin delivery, first developed in AutoPen (44), and this mechanism was also present later on in 2010 in GensuPen^®^ and in 2017 in GensuPen^®^ 2 insulin pens (27, 45). Such a mechanism ensured patients about proper insulin administration, simplified the way of injection, and was convenient for elderly patients (27). Moreover, it was proven that the GensuPen^®^ 2 injector in comparison to NovoPen^®^ 4 (Novo Nordisk, Bagsværd, Denmark) and HumaPen Ergo^®^ (Eli Lilly, Indianapolis, IN) requires reduced force for insulin administration, especially at high doses of the drug (46).

In recent years, further improvement in insulin pen function has been made and there are several ones which possess the memory function of the last dose taken. In 2007, Eli Lilly released the world’s first digital insulin pen with memory function, namely, HumaPen Memoir (47). Soon after, in 2010, Novo Nordisk launched NovoPen^®^ Echo (48), the first insulin pen with memory function and half-unit dosing feature. Most of the insulin pens available in the market have the feature to deliver insulin in 1-unit increments, and only a few deliver in half-units. 0.5-increment insulin pens are designed for patients who need small insulin doses, and the available ones are HumaPen Luxura HD, Humalog^®^ Junior KwikPen^®^, NovoPen^®^ Demi, Junior, Echo, JuniorSTAR^®^, and InPen™. Based on the trials’ outcomes, children, adolescents, and their parents appreciated both the memory function and simplicity of junior devices (49, 50).

Cited studies related to reusable insulin pens are summarized in **Table 1**, and the technical characteristics of reusable insulin pens are presented in **Table 2**.

**Table 1:** Reusable insulin pens.

Study, year	Device studied/device compared	Type of insulin	Participants	Study design	Results
Berger et al., 1985 (31)	NovoPen^®^	Short-acting human insulin (Actrapid HM)	16 adults (10 females, 6 males) aged 21–45 years with T1DM	6-week randomized, controlled, crossover study. During the first treatment period (3 weeks), the patients were instructed to take short-acting insulin with the new device and during the next 3 weeks to take the insulin with their conventional syringes. Intermediate/long-lasting insulin was taken with usual syringes in both study periods.	No significant differences (p > 0.05) in blood glucose profile, HbA1c, and hypoglycemia frequency were found between syringes and new device use. 14 patients found that the new device made their life easier.
Saurbrey et al., 1985 (51)	NovoPen^®^	Short-acting insulin (Actrapid HM)	16 adults (10 females, 6 males) aged 21–45 years with T1DM	10-month follow-up study of the study by Berger et al.	15 patients were still using the NovoPen^®^. There were no differences in mean blood glucose, HbA1c, and number of hypoglycemia (p > 0.05). No significant difference between HbA1c values was found between the outcomes after 6 weeks and 10 months of NovoPen^®^ use.
Jefferson et al., 1985 (32)	NovoPen^®^	Short-acting, human insulin (Actrapid HM)	11 adolescents (7 boys, 4 girls) aged 12–16 years with T1DM	3-month observational study. During 4 weeks of the run-in period, the patients were prepared to the study by optimizing the blood glucose levels, and in the end of the fourth week, the therapy was changed from a conventional to multiple-injection regimen (MIR). Next, the patients started a 3-month observation of the MIR treatment with NovoPen^®^ and a single injection of Human Monotard insulin using a conventional syringe.	10 patients completed the study. There was a non-significant reduction of HbA1c. Moreover, mean blood values were lowered but only in pre-lunch measurements were significantly reduced (p < 0.02). Greater flexibility of timing and size of meals was an overriding advantage of NovoPen^®^ use in the final interview.
Walters et al., 1985 (52)	NovoPen^®^	Short-acting, human insulin (Actrapid HM)	31 patients (20 males, 11 females) aged 16–57 years with T1DM	48-week observational study. After 4 weeks of run-in period, the participants started therapy with MIR using NovoPen^®^ with one injection of Human Monotard from the usual syringe.	27 patients completed the study. Reduction of mean HbA1c values was observed (11.5% in week 0 vs. 10.3% in week 48, p < 0.01). In the final interview, the device was well accepted and 27 patients would like to continue the treatment with NovoPen^®^.
Dahl-Jorgensen et al., 1986 (33)	NovoPen^®^	Short-acting, human insulin (Actrapid HM)	10 adults (5 males, 5 females) aged 21–34 years with IDDM.	6- to 9-month observational study. Patients who had used MIR therapy from conventional syringe for a minimum of 1 year previously started using NovoPen^®^ for short-acting insulin injections (Actrapid HM). A single injection of NPH insulin (Insulatard) was maintained.	HbA1c increased during the pen injector treatment (from 8.8% to 9.3%; p < 0.01). All but one patient had technical problems with NovoPen^®^. All participants desired to continue using the pen injector because of the simplicity of the device and greater flexibility of meal time.
Jensen et al., 1986 (53)	NovoPen^®^	Short-acting, human insulin (Actrapid HM)	20 adults (11 males, 9 females) aged 19–53 years with IDDM.	24-week observational study. Study started with 8 weeks of run-in period. Next, the patients started multiple injections insulin therapy with NovoPen^®^ and a single injection of intermediate-acting insulin (Protaphane) from the conventional syringe.	HbA1c improved during the study (from mean 8.7% to mean 7.9%; p < 0.05). The frequency of hypoglycemia was significantly reduced during the training period (from 1.2 attack/patient/week to 0.3 attack/patient/week; p < 0.01).
Jorgensen et al., 1988 (38)	Insuject-X (NovoPen^®^ 2)	Intermediate-acting NPH-insulin (Insulatard Human)	50 adults (28 males, 22 females) aged 18–56 years with IDDM	6-month randomized, control, crossover trial. All participants were using MIR of soluble insulin (Velosulin Human) from pen injector (Insuject) with a single injection of NPH insulin (Insulatard Human) from the conventional syringe before the study. The first group was continuing multiple injections with the pen injector and NPH insulin from the conventional syringe in the first 3 months of the trial. In the next study period, the group started to administer NPH insulin (Ultratard Human) in the pen injector Insuject-X. The second study group started the trial in the reverse order.	No differences in the metabolic control were found between both study groups. In the final questionnaires, 86% of the patients found the NPH pen injector less complicated to use than usual syringes. All but 2 patients wished to continue using Insuject-X in the future.
Murray et al., 1988 (34)	NovoPen^®^	Short-acting, human insulin (Actrapid HM)	78 adults (44 females, 34 males) aged 18–60 years with T1DM	20-week randomized, controlled trial. After a 6-week run-in period of twice-daily injections with fast and intermediate-acting insulin, patients were randomized into 2 groups. One of them (37 patients) was continuing the two-step insulin regimen with usual syringes. The second group (41 patients) started another regimen with 3 times daily injections of Actrapid made with NovoPen^®^ and a single injection of ultralente insulin (Ultratard).	No significant differences (p > 0.05) in blood glucose profile, HbA1c, and frequency of hypoglycemia were found between the study groups. Patients presented a high level of satisfaction with NovoPen^®^ for the effect on lifestyle (78%) and increased flexibility (81%). 95% of patients preferred using NovoPen^®^ than conventional syringes. In a questionnaire before the study, 47% of the participants revealed that a rigorous daily schedule for meals and activity was the most important disadvantage. At the end of the study, only 21% and 10% respectively still considered these problems as inconvenient. Moreover, patients expressed greater flexibility of meal times and all but one wanted to continue MIR with NovoPen^®^.
Saurbrey et al., 1988 (54)	NovoPen^®^	Short-acting, human insulin (Actrapid HM)	21 adult patients (9 females, 12 males) with T1DM	20-week randomized, controlled, crossover trial. Comparison of intensified conventional treatment (ICT) with continuous subcutaneous insulin injection (CSII). In the first study period (10 weeks), the patients were treated with MIR using NovoPen^®^ with Actrapid insulin plus a single injection of intermediate-acting insulin (Monotard HM). In the next 10-week period, the participants were treated by CSII with a Medix or Auto-Syringe pump.	19 patients completed the study. HbA1c declined significantly in both groups with no differences between the responses (ICT 7.6%; CSII 8.7%). Mean blood glucose was slightly lower in CSII (p < 0.05). There were no differences in frequency of hypoglycemia between ICT and CSII. In the questionnaire, all patients found NovoPen^®^ is better than conventional therapy. Moreover, 12 patients would choose ICT with NovoPen^®^ and 6 ones CSII for the future treatment.
Houtzagers et al., 1989 (55)	NovoPen^®^	Short-acting, human insulin (Actrapid HM)	16 adults (11 males, 5 females) aged 18–63 years with T1DM.	48-week randomized, controlled, crossover trial. Study started with an 8-week run-in period after which patients were included to 2 study periods lasting 24 weeks each. Participants were allocated randomly in one of the study groups: twice-daily syringe injections with human short-acting (Actrapid HM) and intermediate-acting isophane insulin (NPH; Protaphane HM) or 3 times daily preprandial injections of human short-acting insulin (Actrapid HM) with a single injection of human ultralente insulin (Ultratard HM).	The mean daily home blood glucose concentration was significantly lower in the pen-injector group (7.1 ± 0.4 vs. 8.2 ± 0.5 mmol I^-l^, p < 0.05). Neither HbA1c nor fructosamine outcomes did not differ between the syringe and pen injector groups. At the end of the study, 13 patients decided to continue the MIR with NovoPen^®^.
Houtzagers et al., 1989 (56)	NovoPen^®^	Short-acting, human insulin (Actrapid HM)	16 adults (11 males, 5 females) aged 18–65 years with T1DM	12-month randomized, controlled, crossover trial. Following an 8-week run-in period, participants were randomly allocated to twice daily injections of combined human short-acting (Actrapid HM) and intermediate-acting isophane (NPH) insulin (Protaphane HM) with a conventional syringe or administration of human short-acting insulin (Actrapid HM) in 3 preprandial injections from NovoPen^®^ with a single-syringe injection of human ultralente insulin (Ultratard HM).	HbA1c was not significantly different in both study groups (8.2 ± 0.4 vs. 7.6 ± 0.4%). In the questionnaires completed at the end of the study periods, the patients using the pen injector presented significantly less state anxiety (p < 0.05) and tended to experience a better self-concept as having diabetes (p < 0.06).
Tallroth et al., 1989 (57)	NovoPen^®^	Short-acting insulin (Actrapid HM)	18 adults (16 males, 2 females) aged 31.0 ± 7.4 years with T1DM	6-month randomized, controlled, crossover trial. Patients were randomly allocated into group A or B. Group A started a 3-month study period with premeal injections of short-acting insulin with NovoPen^®^ and intermediate-acting insulin with ordinary syringes. In the following 3 months, the therapy was continued with three daily insulin injections of intermediate- and short-acting insulin from conventional syringe. Group B participated in the study in the reverse order.	Both groups expressed improved mood and well-being in general during multiple insulin injections. Moreover, increased experience of freedom and less content meal times during pen injector treatment were noted. Metabolic control outcomes differ significantly neither in group A nor B after 6 in the end of the study.
Tubiana-Rufi et al., 1989 (58)	NovoPen^®^	Short-acting, human insulin (Actrapid HM)	15 adolescents (8 boys, 7 girls) aged 5–19.5 years with IDDM	6- to 24-month observational study. Patients, previously treated with 2 daily injections of mixed insulin, started the therapy with multiple injections of short-acting human insulin (Actrapid HM) using NovoPen^®^ before each meal. A single dose of long-lasting insulin (Ultratard HM) was injected separately with the conventional syringe.	Significant improvement in metabolic control was observed in the insufficiently controlled group of patients (n = 8) where HbA1c decreased from 8.4 ± 1.8% to 7.3 ± 1.2% (p < 0.05) in the first 6 months of NovoPen^®^ therapy. No more metabolic improvement was observed. The long-term acceptability of multiple injections with NovoPen^®^ was excellent; 100% patients experienced the pen injector as a progress, and 80% would like to continue the treatment in the future.
Engstrom, 1990 (39)	NovoPen^®^	Intermediate-acting insulin NPH (Protaphane HM)	40 patients with IDDM	24-week randomized, controlled, crossover trial. Before the study, all participants were treated with multiple injections of short-acting insulin with the pen injector and single injection of basal NPH insulin from the conventional syringe. In the first 12 weeks, one group started using NovoPen^®^ to inject NPH insulin and the second one continued using usual syringes to administer isophane insulin. The second period was followed in the reverse order.	Outcomes of metabolic control were similar in both study groups. Total soluble insulin doses were significantly higher (31.3 vs. 29.9 U/day, p = 0.02), similarly the ones before breakfast (11.1 vs. 10.6 U/day, p = 0.04) when NovoPen^®^ with NPH insulin were used. All but one patient found it easy to resuspend the isophane insulin in the penfill and was confident in the dose accuracy. 38 (of 40 patients) decided to continue using NovoPen^®^ for basal insulin injections.
Henderson et Tindall, 1990 (40)	NovoPen^®^ 2	Premixed insulin (Actraphane)	32 patients with IDDM	3-month observational study. Two groups took part in the trial: volunteers testing NovoPen^®^ II [12 patients (9 males, 3 females)] and the ones continuing twice daily injections with conventional syringe [20 patients (12 males, 8 females)]. The NovoPen^®^ II group completed the quality-of-life (QoL) questionnaire at the beginning of the trial and 3 months later while the control group filled in the one 3 times (to test the reliability of the survey): during the first visit to clinic, 2 weeks later, and at the end of the study.	67% of patients found the NovoPen^®^ II easy to use, but only half found it more convenient than usual syringes. No significant differences were found in the questionnaire outcomes between the study groups—NovoPen^®^ II did not markedly alter patients’ QoL.
Kadiri et al., 1998 (59)	NovoPen^®^ 3	Intermediate-acting insulin NPH (Insulatard HM) or premixed one (Mixtard HM)	96 adults with NIDDM	24-week, open, randomized, crossover trial. Patients with NIDDM and secondary failure (fasting blood glucose > 7.8 mmol/l and HbA1c >25% above the upper limit). All patients were treated with OHAs and diet for at least 1 year before entering the study. The trial consisted of two 12-week periods of insulin administration. Group A started with NovoPen^®^ 3 in Period 1 and crossed over to syringe/vial use in Period 2. Group B followed the study in the inverse order.	78 patients completed the study. Pain during injections was significantly reduced in the NovoPen^®^ 3 periods (p = 0.0018), including patients in group B who reported lower injection pain using NovoPen^®^ 3 after syringes/vials (p = 0.0003). Acceptance of the injections was significantly higher in the NovoPen^®^ group (p = 0.0059). 89.5% of patients preferred NovoPen^®^ 3 to syringes and vials.
Stocks et al., 2001 (43)	HumaPen^®^ Ergo vs. NovoPen^®^ 3 and vial/syringes	Intermediate-acting insulin NPH or premixed 30/70 one	70 insulin-requiring patients (aged 13–65 years, mean 44.6) with T1DM and T2DM	5-7 week, multicenter, observational study. Patients administering insulin at least 3 months prior to study entry were asked to answer the questionnaire to assess the level of satisfaction with their current delivery device. Next, participants were instructed how to use HumaPen^®^ Ergo and started injecting insulin in their previous regimen with the new injector. After 5–7 weeks, in the end of the study, patients were asked to answer the questionnaire regarding the acceptability of HumaPen^®^ Ergo, compared with their previous devices.	>70% of both syringe and NovoPen^®^ 3 users rated HumaPen^®^ Ergo as easy to use in all aspects. The main advantages of the new device were ease of holding during injection, possibility of correcting the doses and the procedure of cartridge changing. At the end of the study, 74% of syringe users and 72% of previous injector users decided to continue administering insulin with HumaPen^®^ Ergo.
Ristic et al., 2002 (35)	HumaPen^®^ Ergo	Intermediate-acting insulin NPH or premixed 30/70 one	230 patients with T1DM (23%) or T2DM (73%) and 24 HCPs	5- to 7-week multicenter, observational study (consisted of two open-label studies with identical design). Participants who were using another injector before the study started the insulin administration with HumaPen^®^ Ergo. The visits took place in the beginning of the study, after the next 3 weeks, and again in the 7th week of the study. The acceptability of the HumaPen^®^ Ergo was evaluated with a questionnaire in the end of the trial. The HCPs assessed the pen injector with the same criteria as the patients.	Participants considered HumaPen^®^ Ergo as easy/very easy in learning to use (97%), reading the dose (95%), correcting the dose (97%), and holding during injection (62%). Most of patients (Study 1/2: 89%/93%) found the pen easier/much easier to correct the dose than the previously used injector. 60%/69% of the study group would continue using HumaPen^®^ Ergo and recommend the model to the others HCPs and would recommend the injector because of the ease in dialing back with no insulin waste (80%) and reading the dose (74%).
Summers et al., 2004 (21)	Insulin injection pen device (IIPD) vs. vial and syringe	N/A	242 respondents with T1DM and T2DM (99 insulin users and 143 insulin nonusers) aged 18–83 years (mean 53.4 ± 13.2 years)	US residents completed an email survey with a 19-item self-administered questionnaire. Items were designed to evaluate patients’ experience with IIPD and vial and syringe. The results were analyzed on a 5-point Likert-type scale. Higher scores mean greater agreement. The survey examined ease of use, activity interference, and social acceptability of IIPD and vial and syringe.	Overall preference for the IIPD was higher than that for vial and syringes among both groups (insulin users and nonusers), mainly because of social acceptability. However, current insulin users claimed that social acceptability and ease of use were the most significant predictors of preference vial and syringes. For insulin non-users, these preference predictors were activity interference and also ease of use.
Larbig et al., 2005 (44)	AutoPen^®^ 24	N/A	40 adults (20 men, 20 women mean aged 49.3 ± 15.1 years), 20 patients with T1DM and 20 ones with T2DM	6-month multicenter, open, randomized, crossover study. Before the study, the patients were trained to handle the insulin pens properly. Group A started the study with AutoPen^®^ 24 and after 3 months switched to OptiPen^®^ Pro. Group B followed the study in the reverse order. All the patients participated in all three visits every 12 ± 2 weeks each. After every study period, the patients completed a standardized patient experience and preference questionnaires.	Both groups presented similar metabolic control and number of hypoglycemic episodes. AutoPen^®^ 24 presented a high level of acceptance in patients (in comparison with OptiPen^®^ Pro) and was preferred by older patients with T2DM.
Goksen et al., 2006 (60)	OptiPen^®^ Pro-1	NPH insulin	32 patients (mean age 17.0 ± 4.4 years) with T1DM	6-month observational study. Patients were treated with NPH insulin for at least 6 months before the study. In the beginning of the trial, they were transferred to glargine insulin administered with OptiPen^®^ Pro-1. After 6 months of observations, the patients were asked to complete an inquiry form and rate the OptiPen^®^ Pro-1 on a scale (0 = worst, 5 = best).	Patients rated the pen as 5 (9% of patients), 4 (38.4%), 3 (26.4%), 2 (11.7%), 1 (8.8%), and 0 (2.9%). Leakage from the injector was noted in 58.8% of subjects, and 38.2% of the ones reported a problem with a dosage button (it was not locking when it was fully depressed after the injection). 61.7% of patients exchanged the pen for an insulin syringe or insulin detemir.
Venekamp et al., 2006 (61)	HumaPen^®^ Memoir	Lispro insulin (Humalog^®^) and human NPH one	300 participants (aged 18–75 years) with T1DM (38%) or T2DM (62%).	6- to 10-week multicenter, open-label, single-arm study. The study involved 3 office visits in 6–10 weeks. Patients (who were regularly using pen injectors prior the study) started injections of basal/prandial doses with HumaPen^®^ Memoir. Moreover, patients were recording any complaints that they had during the trial. The complaints were categorized as functional or non-functional. Participants had a possibility to call the investigators if any help with the injector was needed during the study.	287 patients completed all 3 visits. There were 33 (10.5%) non-functional and 24 (7.6%) functional complaints reported (15 user-related and 8 electronic failures), but none of them resulted in a serious adverse event. No pen-related hypoglycemia and 2 pen-related hyperglycemias were reported. 81.4% of participants preferred the HumaPen^®^ Memoir than their recent injectors.
Olsen et al., 2010 (49)	NovoPen^®^ Echo	N/A	205 participants (79 children aged 7–18 years with T1DM, 78 parents and 48 HCPs).	Observational study. Participants were asked to assess the usability of the device they were using before and the NovoPen^®^ Echo. Firstly, they completed specially designed tasks (setting up the pen, adjusting and injecting a dose, operating the memory function and subjective assessment). Afterward, participants filled up rating scales (1 = most favorable; 6 = least favorable) to rank each pen.	NovoPen^®^ Echo was highly rated for the design and overall appearance (1.71 ± 0.79) in comparison with NovoPen^®^ Junior (2.02 ± 0.93) and HumaPen^®^ Luxura HD (2.36 ± 1.01). Moreover, 94% parents and 89% children/adolescents found the memory function very easy/easy to use. 80% participants preferred NovoPen^®^ Echo to the other pens (p < 0.0001).
Israel-Bultman et al., 2011 (62)	NovoPen^®^ 4	Human insulin or analogues	1854 adults with T1DM or T2DM	12-week, open-label, observational study. The study investigated the preference of NovoPen^®^ 4 usage among patients who previously administered insulin with other pen injectors (NovoPen^®^ 3, HumaPen^®^ Ergo, OptiPen Pro). During the first visit, participants completed the Investigator’s Questionnaire and received a NovoPen^®^ 4 with a complete instruction on how to use it. Moreover, patients’ satisfaction with the previous treatment was analyzed with validated DTSQ. In the final visit (after 12 weeks), the new treatment was evaluated and patients completed the Investigator’s Questionnaire again.	Patients’ satisfaction improved from 26.5 to 30.5 in DTSQ score (p < 0.0001). 83.3% of patients found NovoPen^®^ 4 easier to use overall (p < 0.0001), and over 70% of them declared that the new device was less complicated to set, read, correct, inject, and change the cartridge than in the previous injectors. 97.2% of healthcare professionals would recommend the NovoPen^®^ 4 to the other patients.
Sommavilla et al., 2011 (42)	NovoPen^®^ 4 vs. NovoPen^®^ 3	N/A	117 participants: 82 current NovoPen^®^ 3 users (mean age 48.5 ± 1.6 years) and 34 insulin-naïve patients (mean age 61.8 years ± 1.9) with T1DM or T2DM	Multicenter, open-label, crossover study. In the first step of the study, the group of patients currently using NovoPen^®^ 3 were asked to handle NovoPen^®^ 4 and complete a sequence of tasks within 5 min. The second, crossover part of the trial concerned both groups of patients (NovoPen^®^ 3 users and insulin-naïve patients). The first half of every group received a time-recorded training about using NovoPen^®^ 3 before completing a series of tasks. In the end of the tasks, the patients were asked to evaluate handling the device in a questionnaire. In the second step, the participants completed the same sequence of tasks with another device—NovoPen^®^ 4. The other half of the study groups assessed the injectors in the reverse order.	Current NovoPen^®^ 3 users completed the tasks with NovoPen^®^ 4 in an average time of 1.94 min (range, 0.57–4.98 min). Survey responses presented less difficulty and more confidence in handling NovoPen^®^ 4 than NovoPen^®^ 3 in both groups. 96.3% NovoPen^®^ 3 users and 100% insulin-naïve patients preferred to use NovoPen^®^ 4 (p < 0.0001).
Klonoff et al., 2013 (50)	JuniorSTAR^®^	N/A	167 participants (nurses working with children with T1DM, children/adolescents with T1DM and their parents)	Observational study. In the study, the following participated: 109 nurses working with children with T1DM; 16 parents of children aged < 5 years; 8 children aged 6–12 years; 12 parents of children aged 6–12 years and 22 adolescents aged 13–18 years. Participants were asked to assess the JuniorSTAR^®^ pen injector on 3 five-point scales: - when rating the product: 1 = very poor; 5 = very good or 1 = very difficult; 5 = very easy, - when asked to agree/disagree: 1 = completely disagree; 5 = completely agree. Positive response means a percentage of either a 4 or a 5 score.	98% of the study population found that the insulin injector helped patients achieve a high level of dose dialing accuracy (93% of children/parents and 100% of nurses). The key advantages of the JuniorSTAR^®^ (found in at least 84% of all participants) are practicality, ease of carrying (84%), ease of reading the dose (96%), ease of dialing back (87%), and a suitable injection force (87%). When the respondents were asked to describe the pen in one word, the most common replies were as follows: practical, easy, and simple.
Grabner et al., 2013 (18)	Pen vs. vial	Glargine insulin	2,531 insulin-naive patients with T2DM (1384 pen and 1147 vial users)	Retrospective, observational cohort study. Patients were included into the study using data from HealthCore Integrated Research Database. Patients were treated with at least 1 oral antidiabetic or glucagon-like peptide-1 receptor agonist (GLP-1) at baseline. The observations were provided 6 months before first insulin use (first insulin prescription) and 12 months later (follow-up period). The analysis covered 1-year outcomes including treatment persistence and adherence, HbA1c, hypoglycemia rates and healthcare costs.	Patients initiating insulin therapy with pens (glargine) were more persistent (60.6% vs. 50.1%, p < 0.001), adherent (medication possession ratio, 0.73 vs. 0.57, p < 0.001) and with lower HbA1c levels in follow-up (mean adjusted change, -1.05 vs. 0.73, p < 0.001) in comparison to vial patients. In both cohorts, hypoglycemia occurred at similar rates (3.8% vs. 5.2% respectively, p = 0.21). Study drug costs were higher among pen users ($1164 vs. $762, p < 0.001).
Asche et al., 2013 (15)	Pen vs. vial	Aspart insulin	11,588 adults patients from the MarketScan database (6,065 pen users and 5,523 vial ones) and 8,294 adults from the LifeLink database (4,512 pen users and 3,782 vial ones) with T2DM and T1DM	Longitudinal retrospective analysis based on the MarketScan and IMS LifeLink databases. Study groups contained patients initiating treatment with insulin aspart administered by pen or vial and syringe. The data were collected based on outpatient pharmacy claims data. During the 12-month post-index period, patients had at least 2 claims for the index treatment.	Vial and syringe use was characterized by 35% greater odds of at least one hypoglycemic episode than pen use (p < 0.001) in the MarketScan database and 44% greater odds in the LifeLink database (p < 0.001). Use of vial and syringes was associated with 89% and 62.7% (respectively, both p < 0.001)) greater healthcare costs because of hypoglycemic events than use of pens.
Ahmann et al., 2014 (20)	Pen vs. vial	Glargine insulin	405 insulin-naïve adults with T2DM (aged 18–85 years)	Randomized, open-label, crossover study. Patients received basal insulin (glargine) in one of two treatment sequences (2 weeks of using pen followed by 2 weeks of using vial and syringe or vice versa). Patient device preference was evaluated by the Insulin Injection Preference Questionnaire in the first end point (at week 4—the end of the crossover period). Then, patient preference and HCP recommendation were assessed with one global item and 3 others (blood glucose control, reluctance to use insulin, long-term insulin use) using a 5-point scale (1 = not preferred, 5 = preferred/recommended). Next, patients were re-randomized to pen or vial and syringe group for further observation (6, 10, and 30 weeks) to evaluate clinical end-points (HbA1c, fasting blood glucose levels) and safety outcomes (hypoglycemia, adverse events).	Pens were preferred by patients and strongly recommended by HCPs over vials and syringes (p < 0.001). Corresponding responses were observed by both groups (patients and HCPS) in the three subscale items. Fasting glucose levels, HbA1c levels, and hypoglycemia rates were comparable in both pen and vial/syringe users.
Lasalvia et al, 2016 (23)	Pen vs. vial	Glargine, detemir, NPH, aspart, premixed human 30/70, lispro	Study groups generally composed of adults with T2DM.	Meta-analysis. 10,348 articles from 8 different databases, of which 17 studies were selected: 7 experimental and 10 analytical. Studies concerned a comparison of insulin administration by pen devices with vial and syringes. HbA1c, hypoglycemia, adherence, persistence, patient preference, and QoL were analyzed.	Pen devices presented better results in mean HbA1c change, frequency of hypoglycemia, adherence, and persistence in comparison with vial and syringes. Among patients with good metabolic control (HbA1c < 7%) no difference was observed. Tendency to prefer pen devices was observed, however unvalidated tools were used in the analysis.
Gorska-Ciebiada et al., 2020 (36)	GensuPen^®^	Short- and long-acting insulins, premixed human 30/70, 40/60 and 50/50 ones	4,513 adults (mean age 65.3 ± 10.2 years) with T2DM	12-week, multicenter, observational trial EGIDA II (Education and GensuPen^®^ In Diabetology II) Participants were divided into 2 groups: A—treated with GensuPen^®^; B—treated with other pens. Before the study, all the subjects were educated by trained HCPs. Patients were asked to complete the questionnaires regarding injection parameters, pain scale, and satisfaction of the treatment before (visit 1) and after the study (visit 2).	Patients’ utility, comfort, and satisfaction with the treatment increased, wherein group A presented a greater increase. In both study groups, mean glucose levels (from self-control diaries) were significantly lower after 3 months of the trial, but group A presented a greater difference between visits 1 and 2. In both groups, a significant decrease in sensation of pain was observed, with a greater decrease in group A. Moreover, education of the patients could help to improve the metabolic control and technique of insulin injections, reduce BMI and pain sensation.
Masierek M et al., 2020 (27)	GensuPen^®^	Gensulin^®^ R, Gensulin^®^ N, and premixed insulins M30, M40, and M50	10,309 adults (mean 63.3 ± 12.0 years) with T2DM	4-week multicenter, prospective, observational, open-label study. The trial consisted of one visit in the office (during study enrolment) and two telephone contacts (performed 7 days after enrolment and 4 weeks ± 7 days later). All patients were educated about the proper use of GensuPen^®^ and maintained on Gensulin^®^ (Gensulin^®^ R, N or premixed M30, M40, M50). Moreover, participants had an opportunity to contact dedicated helpline in case of any technical problems with the injector. During the first telephone contact, patients were asked about any problems and needed information regarding GensuPen^®^ use. The next call (after the study) was aimed at assessing patients’ safety and comfort concerning GensuPen^®^. The interview was based on two questionnaires concerning evaluation of the GensuPen^®^ and comparing the new injector with previously used ones (if applicable).	GensuPen^®^ was rated as very good in confirmation of successful administration (92.0%), setting a dose (87.8%), trigger location (80.9%), and injection force (75.0%). Adverse events occurred in 0.6% of participants and none was serious. Moreover, the overall safety of the device was rated as high (severe hypoglycemia affected only 0.2% of the study group).
Boye et al., 2021 (25)		N/A	504 adults (251 UK, 253 US) treated with injections of insulin (49.6%) or GLP-1 receptor agonist (50.4%)	Observational, online survey study. Patients treated with insulin or GLP-1 receptor agonist were presented with a list of 17 characteristics of injectable medication and ask to indicate which were most important for them.	The most frequently selected characteristics were confidence in administering the correct dose (n = 300, 59.5%); ease of selecting the correct dose (n = 268, 53.2%); overall ease of using the injection device (n = 239, 47.4%); frequency of injections (n = 223, 44.2%); ease of carrying the device when necessary to inject away from home (n = 190, 37.7%). Respondents least often chose dose escalation (n = 79, 15.7%); handling the needle (n = 74, 14.7%); connectivity to an electronic device (n = 70, 13.9%); and the time required to prepare and inject each dose (n = 62, 12.3%).

DM, diabetes mellitus; T2DM, type 2 diabetes mellitus; GLP-1, glucagon-like peptide 1; IIPD, insulin injection pen device; DTSQ, Diabetes Treatment Satisfaction Questionnaire. N/A, not applicable.

**Table 2:** Characteristics of reusable insulin pens.

Pen device	Type of insulin/company	Dose range (dose increment)	Memoir	Dialing forward and backward without wasting insulin	Special characteristics
NovoPen^®^ 3 (63)	Novo Nordisk 3-ml cartridges	2–70 units (1 unit)	No	No	N/A
NovoPen^®^ 1.5 (41)	Novo Nordisk 1.5-ml cartridges	1–40 units (1 unit)	No	No	Shorter device.
NovoPen^®^ 3 Demi (64)	Novo Nordisk 3-ml cartridges	1–35 units (0.5 unit)	No	No	First 0.5-unit increment pen.
NovoPen^®^ Junior (65)	Novo Nordisk 3-ml cartridges	1–35 units (0.5 unit)	No	No	Vibrant colors.
NovoPen^®^ 4 (66)	Novo Nordisk 3-ml cartridges	1–60 units (1 unit)	No	Yes	Audible confirmatory dosing click. Safety feature preventing selection of a dose greater than the amount of insulin left in the cartridge.
NovoPen^®^ Echo (67)	Novo Nordisk 3-ml cartridges	0.5–30 units (0.5 unit)	Yes	Yes	Two color variants and choice of skins available. Electronic display showing the last dose of insulin administrated.
NovoPen^®^ 5 (68)	Novo Nordisk 3-ml cartridges	1–60 units (1 unit)	Yes	Yes	2 color variants, electronic display showing the last dose of insulin administrated.
AutoPen 24^®^ (69)	Sanofi Aventis 3-ml cartridges	1–21 units (1 unit) or 2–42 units (2 units)	No	No	Side-mounted release button.
AutoPen^®^ Classic (70)	Eli Lilly or Wockhardt 3-ml cartridges	1–21 units (1 unit) or 2–42 units (2 units)	No	No	Side-mounted release button.
AutoPen 2^®^ (71)	N/A	1–72 units (1 unit)	No	Yes	Side-mounted release button. Dose correction button. Identity rings for different types of insulin.
OptiPen^®^ Pro 1 (72)	Sanofi-Aventis 3-ml cartridges	1–60 units (1 unit)	No	Yes	Digital display to set the insulin dose.
OptiPen^®^ Pro 2 (72)	Sanofi-Aventis 3-ml cartridges	2–60 units (2 units)	No	Yes	Digital display to set the insulin dose.
HumaPen^®^ Ergo (2002) (73)	Eli Lilly 3-ml cartridges	1–60 units (1 unit)	Yes	Yes	N/A
HumaPen^®^ Ergo II (74)	Eli Lilly 3-ml cartridges	1–60 units (1 unit)	Yes	Yes	N/A
HumaPen^®^ Luxura (75)	Eli Lilly 3-ml cartridges	1–60 units (1 unit)	Yes	Yes	2 color variants.
HumaPen^®^ Luxura HD (76)	Eli Lilly 3-ml cartridges	1–30 units (0.5 unit)	Yes	Yes	N/A
HumaPen^®^ Memoir (77)	Eli Lilly 3-ml cartridges	1–60 units (1 unit)	Yes	Yes	Digital display with time, date and dose of insulin.
OptiClik^®^ (78)	Lantus 3 ml (Sanofi-Aventis) Cartridge System	1–80 units (1 unit)	No	No	Digital display.
BerliPen^®^ 301 (79)	Berlinsulin H or Liprolog 3-ml cartridges	1–21 units (1 unit)	No	No	Side-mounted release button.
BerliPen^®^ 302 (79)	Berlinsulin^®^ H or Liprolog 3-ml cartridges	1–42 units (2 units)	No	No	Side-mounted release button.
BerliPen^®^ Areo 3 (80)	Berlinsulin^®^ H or Liprolog 3-ml cartridges	1–60 units (1 unit)	No	Yes	5 color variants.
GensuPen^®^ (27)	Gensulin^®^ 3-ml cartridges	1–40 units (2 units)	No	Yes	Side-mounted release button. End-of-dose indicator.
GensuPen^®^ 2 (45)	Gensulin^®^ 3-ml cartridges	1–60 units (1 unit)	No	Yes	Side-mounted release button. End-of-dose indicator. 3 color variants
JuniorSTAR^®^ (81)	Lantus^®^, Apidra^®^ or Insuman^®^ (Sanofi-Aventis) 3-ml cartridges	1–30 units (0.5 unit)	No	Yes	3 color variants.
TactiPen^®^ (Itango) (82)	Sanofi-Aventis 3-ml cartridges	1–60 units (1 unit)	No	Yes	4 color variants.

N/A, not applicable.

## Prefilled (Disposable) Insulin Pens

Prefilled (disposable) insulin pens, like reusable ones, are loaded with 3 ml (300 U) of insulin, and some of the patients find it easier to operate than the reusable insulin pens because there is no need to replace the cartridge (83). In 1989, Novo Nordisk launched the world’s first disposable, prefilled insulin pen namely NovoLet^®^ (84) followed by FlexPen^®^ introduced in 2001 (41) and Next Generation FlexPen (NGFP) in 2008 (85) and FlexTouch^®^, a reengineered version of the FlexPen^®^ with a novel injection mechanism, in 2011 (86).

Other prefilled insulin pens include SoloSTAR^®^ (Sanofi) launched in 2008, KwikPen^®^ (Eli Lilly) launched in 2007 (87), and Junior KwikPen^®^ launched in 2017, a half-unit insulin pen (88). Similarly to reusable insulin pens, prefilled ones when compared to vials and syringes were rated as much easier to handle, discreet in public use, confident in proper dose delivery, and preferred by majority of patients (with T1DM and T2DM), healthcare professionals (89–91), and patients’ caregivers (parents, relatives) (92). Moreover, both non-experienced healthcare practitioners and needle-naïve patients found the prefilled insulin pens much easier to teach and learn (93, 94).

For years, insulin pens were used with insulin 100 U/ml, but since the development of higher-concentration insulins, also new insulin pens for 200 and 300 U/ml have been manufactured and used since 2017, namely, Humalog^®^ 200 U/ml KwikPen^®^ (Eli Lilly) (95), Tresiba^®^ 200 U/ml prefilled FlexTouch^®^ (Novo Nordisk) (96), and Glargine U300 SoloSTAR^®^ insulin pen (Sanofi-Aventis) (97). However, we must consider that disposable pens are less environment friendly and this is a globally growing importance nowadays (98). One can just imagine that if a patient is using approximately 40 units of insulin a day there is about 50 prefilled plastic pens thrown away every year and accounting for thousands of patients using insulin pens the number of insulin pens being thrown away per year is accounted in millions. Based just on a small study form Bosnia and Herzegovina published in 2020, it was predicted that only in this small country there were 3.2 million pens used and dispensed annually (99).

Cited studies related to prefilled insulin pens are summarized in **Table 3**, and the technical characteristics of prefilled pens are presented in **Table 4**.

**Table 3:** Prefilled insulin injectors.

Study, year	Device studied/device compared	Participants	Study design	Outcomes
Korytkowski et al., 2003 (89)	FlexPen^®^ vs. vial and syringe	121 adults aged 28–81 years with T1DM and T2DM	8-week multicenter, randomized, open-label, comparative, two-period crossover trial. During the 4-week run-in period, the patients continued the therapy with the previous devices (i.e., their own pens or syringes), to administer a mixture of 70% aspart protamine suspension and 30% aspart insulin. Insulin doses were optimized. Then patients were randomly allocated to one of the study groups. Half of the participants started the trial using prefilled, disposable pens for 4 weeks, and next they were crossed over to a vial/syringe group for another 4 weeks. The second group followed the study in the reverse order. Patients’ preference was assessed based on the Patient Preference Questionnaire in the final visit of the second treatment period.	103 patients completed the study. Most of the patients (78%) preferred the pen over vial and syringe methods, and 85% found the FlexPen^®^ more discreet in public. Ease of pen use was greater for 74% of respondents, and 85% of them considered the insulin dose scale much easier to read in the pen injector. However, metabolic control was comparable in both FlexPen^®^ and vial and syringe group and patients’ HbA1c improved during the study (p < 0.05).
Niskanen et al., 2004 (100)	FlexPen^®^ vs. Humalog^®^ Pen	137 patients (mean aged 62.3 ± 9.2 years) with T2DM	24-week randomized, multinational, multicenter, open-label, 2-period crossover trial. After a 2-week run-in period, patients were randomly involved into a 12-week treatment period with BIAsp 30 (30% of soluble insulin aspart and 70% protaminated insulin aspart) or Mix25 (25% soluble insulin lispro and 75% neutral protamine lispro) using FlexPen^®^ or Humalog^®^ Pen. Next, participants were crossed over to the second treatment period with another type of insulin and pen device. In the final questionnaire, patients’ preference for the pen injectors was assessed.	FlexPen^®^ received the highest rates for all device features assessed in the final questionnaires (all p < 0.005). 32.4% of patients experienced problems with Humalog^®^ Pen when only 9.0% with FlexPen^®^ (p < 0.001). 74.6% of respondents preferred to continue using FlexPen^®^ (in comparison with 14.3% preferred Humalog^®^ Pen, p < 0.001).
Haak et al., 2007 (101)	SoloSTAR^®^, Humalog^®^/Humulin pen, FlexPen^®^, and prototype Pen X	510 patients aged 11–82 years (232 adults with T2DM receiving only OHAs and 278 insulin users with T1DM or T2DM).	Multicenter, observational study The trial consisted of 1-hour face-to-face interviews aimed at evaluating the usability of the devices and patients’ preferences. Firstly, participants were asked to prepare the device and deliver a 40-unit dose relying on their intuition and/or relevant manuals. Any training and maintenance was not provided. Next, respondents evaluated abovementioned procedures for each pen in a five-point scale (1 = poor, 5 = excellent).	Significant majority of patients prepared the SoloSTAR^®^ properly and performed a correct injection with the device in comparison with the other pens (p < 0.05). Moreover, most of the patients (53%) preferred to use SoloSTAR^®^ than Flex Pen^®^ (31%) and Humalog^®^/Humulin pen (15%).
Ignaut et al., 2008 (87)	FlexPen^®^ (NovoLog^®^ Mix 70/30) vs. KwikPen^®^ (Humalog^®^ Mix75/25)	50 insulin pen device (25 FlexPen^®^s and 25 KwikPen^®^s)	In this study, ergonomic features, injection force (as glide force (GF), and glide force variability (GFV)) were measured and compared in FlexPen^®^ and KwikPen^®^ injectors.	FlexPen^®^ was lighter than KwikPen^®^ and had a smaller diameter at the cartridge holder and dose window while KwikPen^®^ presented a shorter overall pen length with a shorter thumb reach at both 30- and 60-unit dose settings. For both the 30-unit and 60-unit doses, maximum GF was lower in KwikPen^®^ than in FlexPen^®^ (3.42 vs. 5.36 lb and 3.61 vs. 5.62, respectively, both p < 0.0001).
Asakura et al., 2009 (93)	FlexPen^®^ vs. vial and syringe	60 HCPs (30 insulin experienced and 30 insulin-naïve ones)	Multicenter, observational study The first part of the study consisted of insulin delivery training among insulin-naïve participants. Next, respondents were randomized into 2 study groups, one of group performed an injection of 10 U with FlexPen^®^ (Day 1) and then with vial/syringe (Day 2). The second group followed the tasks in the reverse order. Subsequently, insulin-naïve HCPs assessed the devices and made an overall comparison in the evaluating questionnaires (rate range: 1 = very poor, 5 = excellent). The second part of the trial depended on the randomized, accuracy testing of the two devices (FlexPen^®^ and vial/syringe) by 30 insulin-experienced and 20 insulin-naïve HCPs. After injecting 10 U of insulin, devices were weighed and the outcomes were converted into insulin units (0.1 g = 10 U).	Insulin therapy-naïve HCPs preferred FlexPen^®^ and found it much easier to handle than vial and syringe (p < 0.001). Moreover, the pen was more accurate than syringe when used by both insulin experienced and non-experienced HCPs (p < 0.001).
Asakura et Jensen, 2009 (102)	FlexPen^®^ vs. OptiClik^®^	61 adults (mean aged 61.9 ± 12.3 years) with T2DM	Randomized, open-label, crossover study. All study groups were insulin-device-naïve. Participants were randomized into intuitiveness and instruction time group and then randomized again to the subgroups starting injections with FlexPen^®^ or OptiClik^®^. The intuitiveness group had to make an injection into a cushion without any training or manual. At the end the study, the group completed a intuitiveness and device understanding questionnaire. The second group received an instruction before injecting a dose. Both groups completed the important features of the device questionnaire. Afterward, everyone received the injectors again and became instructed how to use each pen. In the end, patients fulfilled questionnaires regarding ease of use and overall preference.	FlexPen^®^ required less instruction time and was more intuitive for most of patients (p < 0.001). None in the instruction time group considered FlexPen^®^ difficult to learn, but 45% of the group found OptiClik^®^ difficult/very difficult to learn. Moreover, respondents rated FlexPen^®^ (in comparison to OptiClik^®^) as simpler to use (77% vs. 12%, p < 0.001), easier to inject (67% vs. 13%, p < 0.001), and more convenient 71% vs. 12%, p < 0.001). Analogically, most of the respondents preferred using FlexPen^®^ than OptiClik^®^ (82% vs. 13%, p < 0.001).
Ignaut et al., 2009 (90)	KwikPen^®^ vs. vials and syringes and KwikPen^®^ vs. FlexPen^®^	232 adults (aged 40–75 years) with T1DM or T2DM	1-day, open-label, randomized, crossover study. The study assessed the preference of using KwikPen^®^ vs. vial/syringe and next, KwikPen^®^ vs. FlexPen^®^ among insulin users. Dose accuracy, ease of use (*via* insulin device assessment battery), and respondents’ preference (*via* insulin device preference battery) for each pen were examined, and both pens were evaluated with the final preference questionnaire.	KwikPen^®^ was the most preferable device (over both vial and syringe and FlexPen^®^) because of its appearance, quality, discretion, convenience, public use, ease of learn and use, reliability, dose confidence, and following insulin regimen. KwikPen^®^ was considered as overall the most satisfying device, willingly recommended to others.
Yakushiji et al., 2010 (103)	OptiClik^®^, SoloSTAR^®^, MirioPen, and FlexPen^®^	22 (50% male, 50% female) respondents (11 experienced and 11 non-experienced with insulin injectors) aged 25–57 years.	Observational study Non-experienced participants were educated how to use the injectors. All the respondents made 2 injections with 5 examined devices. The first one was a self-injection in the prosthetic skin attached in the respondents’ flank. The second injection was made to the prosthetic skin placed in the upper arm of the mock patient (other injection). Every injection contained 10 units of saline. In the end, both self- and other injections with every device were evaluated in the questionnaire and rated from 1 to 5.	FlexPen^®^ was rated as the best device for self-injections. However, FlexPen^®^ was also selected the worst one for the other-injections because it was too long, was less stable, and had inadequate visibility of the dial. OptiClik^®^ was evaluated as the best device for other injection but the second worst one to self-injection.
Bailey et al., 2011 (104)	FlexTouch^®^ vs. KwikPen^®^	160 participants: 79 patients with T1DM or T2DM and 81 HCPs (40 physicians, 41 nurses)	1-day, randomized, crossover study. Respondents were randomly assigned to one of the groups (starting the study with FlexTouch^®^ or KwikPen^®^) and then crossover to test the second pen. Participants were trained how to use the devices before the test injections. Next, both patients and HCPs made multiple injections (with randomly altered doses including 20, 40, and 60 U) into a foam cushion and answered questions concerning ease of use, confidence, and preferences.	FlexTouch^®^ (compared to KwikPen^®^) was rated as most preferred device (86% vs. 7%; p < 0.001), easier to use (85% vs. 4%; p < 0.001), and recommended to others (88% vs. 6%; p < 0.001). Additionally, FlexTouch^®^ was characterized as the better device in the injections for ease of depressing the push button and ease of injecting the doses (p < 0.001 for all). FlexTouch^®^ was found as the most confident in correcting and completing insulin delivery (73% vs. 6%; p < 0.001).
Hancu et al., 2011 (105)	SoloSTAR^®^	6481 adults (mean aged 54 years) with T1DM or T2DM	6- to 8-week, multinational, multicenter, open, prospective, observational product/device registry study. At the first, registry visit participants were included to the insulin therapy with the new pens (LANTUS SoloSTAR^®^ and/or Apidra SoloSTAR^®^) and completed a questionnaire regarding their previous experience with insulin injectors (if applicable). Last visit (after 6–8 weeks of SoloSTAR^®^ use) purposed to assess the acceptance of the new disposable pen and compare patients’ experience with the ones used prior the trial. Moreover, series of questions have been asked to evaluate the study period.	6,364 participants were included to the analysis of patient satisfaction. 77.1% patients had used insulin before inclusion in the study. In the trial, SoloSTAR^®^ was used to administer glargine (97.3%) and/or glulisine (36%) insulin. Most of patients found the new disposable injector as “excellent/good” in learning to use (98.3%), ease of use (97.9%), selecting the dose (97.6%), and reading the dose (95.1%). SoloSTAR^®^ was “much easier/easier” for over 80% of the study group (in comparison with previously used pens) because of ease of use (88.4%) and injecting a dose (84.5%). Furthermore, 98% patients desired to continue using SoloSTAR^®^ in the future.
Oyer et al., 2011 (106)	FlexTouch^®^ vs. SoloSTAR^®^	120 participants: - 59 patients with T1DM or T2DM -61 HCPs (30 physicians, 31 nurses)	1-day multicenter, open-label, randomized, crossover study. Respondents were randomly assigned into the study groups (starting test with FlexTouch^®^ or SoloSTAR^®^). Participants were instructed how to use the pen and performed test injections into a foam cushion, dosing 20, 40, and 80 U. In the following step, both study groups were crossed over to test another pen device. Each pen device was assessed separately (in a form evaluating handling and operation of the pen). Moreover, in the final questionnaire respondents completed regarding their preferences.	A significant majority of participants (88%) preferred FlexTouch^®^ over SoloSTAR^®^ (10%). They considered FlexTouch^®^ (vs. SoloSTAR^®^) easier to use (83% vs. 9%), willingly recommended to others (83% vs. 8%; p < 0.001), very/fairly easy to reach the push-button and inject the doses (p < 0.001 for all), more confident in correct insulin delivery (76% vs. 6%; p < 0.001), and managing daily injections (88% vs. 58%).
Buysman et al., 2011 (17)	FlexPen^®^ (Levemir) vs. vials (NPH)	1,876 patients with T2DM (1082 Levemir FlexPen^®^ users and 794 NPH vial ones)	Retrospective analysis from a large geographically diverse US health insurance plan. Patients were divided into 2 study groups—initiating basal insulin therapy with Levemir FlexPen^®^ or NPH in vials. Patients were defined as adherent to therapy if their medication possession ratio (MPR) was at least 80% in the 12-month follow-up period. Patients’ persistence was defined as the lack of gaps in insulin therapy during the follow-up period.	Patients beginning therapy with Levemir FlexPen^®^ had 39% higher adjusted odds of achieving an MPR ≥80% in comparison to patients with NPH vials (OR 1.39, 95% Cl: 0.55–0.70). Moreover, analysis of persistence presented that patients initiating Levemir FlexPen^®^ had a 38% lower hazard of discontinuation compared to NPH vial users (HR 0.62, 95% CI: 0.55–0.70)
Campos et al., 2012 (91)	FlexTouch^®^ vs. vial and syringe	120 participants: - 60 patients with T1DM or T2DM, - 60 HCPs (30 physicians, 30 nurses)	1-day randomized, multicenter, open-label, crossover study. Participants were trained how to use the devices. Next, test injections into foam cushion (dosing 20, 55, and 80 U) were made with both vial and syringe and FlexTouch^®^. Then, respondents separately rated the devices in respect of ease and confidence of use.	FlexTouch^®^ (compared to vial and syringe) was found a preferred device (88% vs. 5%; p < 0.001), easier to use (91% vs. 6%; p < 0.001), and willingly recommended (91% vs. 3%; p < 0.001). Moreover, participants considered FlexTouch^®^ easier to use, more stable during injection, and better in depressing the push-button and reading the dose scale (all p < 0.001). Patients and HCPs using FlexTouch^®^ were also more confident in properly insulin delivery and metabolic control than the ones using vial and syringe (p < 0.001).
Lajara et al., 2012 (94)	FlexTouch^®^ vs. vial and syringe	120 participants: - 30 needle-naïve patients, - 30 vial and syringe-experienced patients, - 30 physicians, - 30 nurses.	1-day randomized, multicenter, open-label, crossover study. All participants received an instruction on how to use the injection device. Then they were asked to make a test injection into a foam cushion (dosing 20, 55, and 80 U) with FlexTouch^®^ or vial and syringe (in a random order) and answer questions on confidence and ease of use (1 = very difficult/not at all confident; 5 = very easy/very confident). In the next step, respondents followed the abovementioned procedures with another device. Finally, all participants completed a preference questionnaire to evaluate both methods.	Both HCPs (nurses: 100% vs. 0%; physicians 87% vs. 7%), needle-naïve (83% vs. 7%), and vial- and syringe-experienced (73% vs. 7%) patients preferred FlexTouch^®^ over vial and syringe for ease of teaching. Moreover, the insulin pen was rated as very/fairly easy for depressing the push-button (physicians: 93% vs. 80%; nurses: 97% vs. 80%; vial and syringe-experienced patients: 93% vs. 90% and needle-naïve ones: 100% vs. 77%).
Nadeau et al., 2012 (107)	FlexTouch^®^ vs. KwikPen^®^ (FT vs. KP) and FlexTouch^®^ vs. SoloSTAR^®^ (FT vs. SS)	FT vs. KP: 160 participants (79 patients with T1DM or T2DM and 81 HCPs) FT vs. SS: 120 participants (59 patients with T1DM or T2DM and 61 HCPs)	1-day, randomized, crossover study. The study consisted of 2 comparison groups: FT to KP and FT to SS. Participants were randomized to start the trial with FT or another pen device and then were crossed over to test the second injector. All respondents were educated about using the devices. Both patients and HCPs were asked to make multiple injections of different doses with each pen (FT vs. KP study: 20, 40, 60 U; FT vs. SS study: 20, 40, 80 U). In the end, participants answered the questions regarding ease of use, learning and teaching, confidence in use, and preference.	FlexTouch^®^ was rated as very/fairly easy to inject, particularly in the maximum dose (compared to KP or SS: ≥80% vs. ≤38% and ≤23%) and very/rather confident in the ability to manage daily injections. FT was also considered as easier to teach and learn to use than KP and SS (all p < 0.001) and preferred for learning and teaching (≥39% vs. ≤4% for KP and ≤6% for SS). Most of the patients and HCPs would recommend FT (≥95%) than KP (≤72%) and SS (≤71%).
Pfutzner et al., 2012 (108)	InnoLet^®^ vs. FlexTouch^®^	90 patients (mean aged 62 ± 8 years) with T1DM or T2DM, with or without impaired dexterity and visual impairment	Patients became stratified into 4 study groups: A—visually impaired with T1DM and impaired dexterity; B—visually impaired with T2DM and impaired dexterity; C—visually impaired with T1DM or T2DM; D—patients without any impairment with T1DM or T2DM. Participants were asked to perform some test injections (dosing 10, 30, and 50 U) and complete a standardized questionnaire assessing the handling of the pen device. The procedure was repeated with a second insulin injector. In the end, patients evaluated the study by completing a comparative questionnaire.	FlexTouch^®^ was preferred in all study groups including 100% of group D (unimpaired patients). Only a few patients with visual/dexterity impairment preferred InnoLet^®^ (group A—13%, group B—3%, group C—14%).
Schipper et al., 2012 (109)	FlexTouch^®^ vs. InnoLet^®^	90 patients (mean aged 62 ± 8 years) with T1DM or T2DM	Patients were assigned to the study groups in random order. Participants (educated how to use the devices) were asked to perform a mock injections (with 10-, 30-, and 50-U doses) and complete a final 41 item standardized questionnaire to assess the device. Patients rated each pen in a five-point scale (1 = very easy, 5 = very difficult) regarding injection confidence and performance, dose setting, general handling, and others.	FlexTouch^®^ (FT) was found better than InnoLet^®^ (IL) for the injection procedure (FT: 1.2 ± 0.1 vs. IL: 2.1 ± 0.4; p < 0.001), general handling (1.3 ± 0.2 vs. 2.3 ± 0.7; p < 0.001), confidence of dosing (1.4 ± 0.2 vs. 2.1 ± 0.9; non-significant). Dose setting was ranked equally (FT: 1.6 ± 0.3, IL: 1.7 ± 0.4, non-significant). 92.2% of patients would recommend FT (IL only 30.0%).
Pfutzner et al., 2013 (92)	FlexTouch^®^ vs. vial and syringe	120 participants: - 40 patients with T1DM or T2DM, - 20 caregivers (i.e. parents, relatives) - 20 physicians, - 40 nurses/certified diabetes educators	1-day single-center, randomized, crossover study. Participants (in random order) were asked to perform testing injections into laboratory tubes (doses of 5, 25, 43, and 79 U) with the devices. Dosing accuracy was measured, and patients completed final questionnaires (device assessment questionnaire, patient perception questionnaire). Next, respondents were crossed over to test another device. At the end of the trial, all participants answered the questions in the device preference questionnaire.	FlexTouch^®^ presented significantly better dosing accuracy when used by all cohorts and at all doses (p < 0.005 for all doses). The pen injector was rated significantly higher than vial and syringe in both device preference questionnaire (93% vs. 2% for vial and syringe; p < 0.001)) and patient perception questionnaire (in all aspects).
Pfutzner et al., 2014 (110)	FlexTouch^®^ (U100 and U200) vs. SoloSTAR^®^	64 adults with T1DM or T2DM and 64 HCPs (32 physicians, 32 nurses)	Multicenter, randomized, open-label, crossover study. The study consisted of one visit. Participants were asked to make 4–6 injections into a foam cushion (dosing 2, 20, 40, 80, 120, and 160 units). Next, they were asked to complete a questionnaire to evaluate the device. These procedures were repeated in each of the three analyzed injectors. After the tests, participants answered the final, overall questions.	Significant majority of participants preferred to use FlexTouch^®^ U100 (93.0%) and U200 (91.4%), even dexterity-impaired and pen-naïve patients in comparison with SoloSTAR^®^ (p < 0.001), respectively.
Cheen et al., 2014 (14)	FlexPen^®^ (NovoMix 30) vs. vial and syringe (Mixtard 30)	955 patients	Retrospective, single-center, longitudinal study. Data were collected from the outpatient clinics database of the largest acute care hospital in Singapore. During 24 months of the observation adherence, compliance (as medication possession ratio - MPR) and persistence were measured, based on electronic medical and pharmacy refill records.	Mean MPR was comparable in vial/syringe and pen users (83.8% ± 26.9% vs. 86.0% ± 23.2% respectively, p = 0.266). Persistent with therapy was higher among pen users (odds ratio = 1.36; 95% CI, 1.01–1.86) after adjusting for sociodemographic and clinical covariates.
Friedrichs et al., 2015 (111)	SoloSTAR^®^ (SS), FlexPen^®^ (FP), KwikPen^®^ (KP), and FlexTouch^®^ (FT 1 and 2)	20 pen-experienced patients (mean aged 55 ± 14 years) with T1DM or T2DM	Patients were asked to dial up from zero to maximum and next, dial down from maximum to zero with each pen. Dialing up and down was recorded with a video, and the torque of the devices was analyzed. Next, 16 pen-experienced people with T2DM rated the subjective comfort for each insulin injector after dialing up and down again.	SS was rated as most comfortable in dialing up by 8 and dialing down by 6 of the 16 respondents; analogically, FP was ranked by 5 and 8, respectively; FT1: 2 and 1; KP: 1 and 1. FT2 was evaluated as least comfortable by 12 and 10 patients. Comfort of up- and down-dialing was considered “very comfortable” for SS by 15 patients each and next, FP (12 and 14), KP (10 each), and FT1 (9 and 7). FT2 was ranked “less/not comfortable” by 10 and 11 respondents, respectively.
Slabaugh et al., 2015 (16)	Pen vs. vial	3,172 insulin-naïve patients with T2DM (aged 18–89 years), 1,231 vial users and 1941 pen ones	Retrospective, observational study. The study analyzed data from Medicare Advantage with Prescription Drug insurance database. Patients initiating basal insulin administration with pens vial/syringes were observed. Persistence and adherence (as proportion of days covered—PDC and medication possession ratio—MPR) were measured during the 12-month follow-up period.	Adjusted mean PDC was significantly higher in the pen cohort than the vial one (0.67 vs. 0.50 respectively, p < 0.001), the same as mean MPR (0.75 vs. 0.57 respectively, p < 0.0001). Adjusted odds for adherence (PDC at least 80%) presented a positive association with insulin pen use (odds ratio = 2.19, 95% CI: 1.86–2.59). The adjusted risk of non-persistence was lower among pen users (hazard ratio = 0.42, 95% CI: 0.38–0.45).
Warren et al., 2019 (112)	FlexTouch^®^ (200 U/ml) vs. SoloSTAR^®^ (100 U/ml)	145 patients with T2DM using ≥ 81 units of insulin a day	32-week randomized, multicenter, open-label, crossover study. Patients became randomly assigned to one of the study groups and started a treatment with insulin degludec (200 U/ml, 3 ml FlexTouch^®^) or glargine (100 U/ml, 3 ml SoloSTAR^®^). After 16 weeks, participants were crossed over to another insulin therapy. Patients’ preference and treatment impact were assessed in the final PRO questionnaires.	Most of the patients found FlexTouch^®^ “extremely easy” for learning (62.5% vs. 43.0%, p < 0.01), maintaining (63.2% vs. 42.2%), and adjusting a dose (63.2 vs. 44.4%). Moreover, respondents considered FlexTouch^®^ (compared to SoloSTAR^®^) as very/extremely confident in using the injector (60.3 vs. 36.3%) and its accuracy (50.7% vs. 30.4%). A significant majority of patients preferred therapy with FlexTouch^®^ (59% vs. 22%), would like to continue (67% vs. 15%), and willingly recommend the injector (67% vs. 14%) in comparison with SoloSTAR^®^.

T1DM, type 1 diabetes mellitus; T2DM, type 2 diabetes mellitus; HCPs, healthcare professionals; MPR, medical possession ratio; U, units of insulin; SS, SoloSTAR^®^; FP, FlexPen^®^; KP, KwikPen^®^; FT1, FlexTouch^®^ 1; FT2, FlexTouch^®^ 2; PDC, proportion of days covered.

**Table 4:** Technical characteristics of prefilled insulin injectors.

Pen device	Type of insulin	Company	Dose range (dose increment)	Memoir	Dialing forward and backward without wasting insulin	Special characteristics
FlexPen^®^	NovoRapid^®^	Novo Nordisk	0–60 units (1 unit)	No	Yes	Compatibility with insulin smart caps
	NovoLog^®^					
	Protaphane^®^					
	Levemir^®^					
	NovoMix 30^®^					
	NovoMix 50^®^					
FlexTouch^®^ 1	Tresiba^®^		0–80 units (1 unit)			
	Ryzodeg^®^					
FlexTouch^®^ 2	Tresiba^®^		0–160 units (2 units)			
KwikPen^®^ Junior	Liprolog^®^	Eli Lilly	0–30 units (0.5 unit)			
	Humalog^®®^					
KwikPen^®^ U-100	Liprolog^®^		0–60 units (1 unit)			
	Humalog^®®^					
	Humulin R^®^					
	Abasaglar^®^					
	Humalog^®^ Mix25					
	Humalog^®^ Mix50					
KwikPen^®^ U-200	Humalog^®^		0–60 units (1 unit)			
KwikPen^®^ U-500	Humulin R^®^		0–300 units (5 units)			
SoloSTAR^®^	Lispro^®^	Sanofi-Aventis	0–80 units (1 unit)			
	Lantus^®^					
	Insuman Basal^®^					
	Insuman Rapid^®^					
	Apidra^®^					
	Toujeo^®^					

## Next-Generation Insulin Pens (Smart Insulin Pens) and Insulin Pen Caps

Nowadays, the way of health delivery is becoming more digital than ever before where face-to-face visits are often replaced by telephone or video contacts and continuous glucose monitoring or glucometer data can be revived through cloud-based data sharing technology which was very pronounced in the COVID-19 era. One of the key problems for patients with T1DM and T2DM treated with multiple daily insulin (MDI) is omitting or late insulin doses which has been found in the study which analyzed data from a continuous glucose monitoring system (CGM) (113). It was also described lately in the study with a Bluetooth^®^-enabled insulin pen cap that all of the patients taking part in the study missed the insulin doses and it could be intentionally missed because of inconvenience or eating pattern or just forgotten (113). It is important to note that it was also calculated already a years ago that omitting only two meal-related insulin doses per week is associated with a 0.4% increase in HbA1c value (114). Another problem with MDI is that patients rely on numeracy skills while deciding about the meal insulin dose, and it has been proven that these skills are many times not good enough which leads to errors in insulin dosing and to poor glycemic control (115–117). Because patients treated with MDI have to make their insulin dosing decisions without access to the amount and timing of previous insulin doses or residual active insulin, this can, on the other hand, cause overlapping of insulin boluses and put a patient at risk of hypoglycemia (118). That is why smart insulin pens and pen caps were and are being developed to overcome these barriers. Information coming from business research indicates that the smart insulin pen market value will significantly increase by the year 2027 in Latin America, the Middle East, and Africa (119) with the greatest market growth in Europe with a trend toward increased use of smart insulin pen market seen also in North America (120).

## Smart Insulin Pens

Smart insulin pens are digital, connected insulin pens which go beyond memory function and automatically transmit information about time and amount of insulin administered to the user’s mobile device and can remind about the insulin dose and help to calculate the bolus (7). The clinical data from the smart insulin pen are transferred wirelessly *via* Bluetooth^®^ technology to an application (app) available for smartphones (7, 121, 122). Therefore, smart insulin pens require the use of an app to collect the data sent from the pen but eliminate the need for manual self-report logbooks (121). Thus, smart insulin pens can help to overcome the challenges that users of pen injectors have to deal with on a daily basis. Smart insulin pens are a relatively new invention, so it should come as no surprise that a few studies have been conducted in this field to date (121). In 2017, the world’s first US Food and Drug Administration (FDA)-approved insulin smart pen which uses Bluetooth^®^ technology, namely, InPen™ (Companion Medical, San Diego, Ca, USA), was launched, and in November 2020 its new version was launched by Medtronic (123). This pen combines the insulin pen with a smartphone app which has the ability to record and store data of insulin injections and recommend doses, as well as display glycemia and related data on the paired smartphone app (124–126). InPen™ is designed for use with rapid-acting insulin U-100 Lilly Humalog^®^ and Novo Nordisk NovoLog^®^ (127). InPen™ is the first of its kind of smart insulin pen that allows to prepare reports for healthcare professionals, reminds about missed doses, and tracks insulin on board, but also alerts the user about an exposure of the device to abnormal (very high or very low) temperatures that may inactivate insulin (124–126, 128). What is likewise important, in InPen™ the dose can be increased or decreased in half-unit steps, and therefore the dose administered is very precise (128, 129). Later on, several new smart insulin pens emerged on the market, namely, ESYSTA^®^ pens (Emperra), Pendiq 2.0 pens (Pendiq), and NovoPen^®^ 6 (Novo Nordisk). It cannot escape the attention that insulin pen injectors may help not only patients but also diabetes care teams. They provide accurate information about missed doses as well as injection times in relation to meals and dose sizes, which is useful in making correct therapeutic decisions and giving personalized treatment plans (121, 130–132). The first study of clinical outcomes using a smart insulin pen was reported in 2020 (133). This investigation was conducted in Sweden and indicated that among patients with T1DM using smart insulin pens, clinical outcomes improved at lower costs compared to standard care. What is even more important, this research suggested that smart insulin pens have the potential to improve glycemic control and decrease glucose variability (133, 134).

## Insulin Pen Caps

Insulin pen caps are another device which does not have a clear definition but displays the quantity of insulin in the pen and integrate the insulin-related information with a mobile app. Insulin pen caps are usually attached to the side or fit in the end of the pen.

A first-of-its-kind smart pen cap for insulin pens (Bigfoot Unity™ Diabetes Management System) launched by Bigfoot Biomedical received FDA clearance in May 2021. This insulin pen cap is integrated with Abbott’s FreeStyle Libre 2 system and translated continuous monitored glucose data into on-demand insulin dose recommendations displayed on the pen cap screen. It is the first and only device which integrates a continuous glucose monitoring system (CGMS) to insulin dose recommendation (135).

Another smart cap integrated with a dedicated mobile app is GoCap (Common Sensing company) (136). The integration with the application helps calculate the meal or correct boluses, preventing overdosing by active insulin display (125, 136). Moreover, individual reminders allow to keep the schedule of basal insulin (136). Similarly, Insulclock^®^ is an electronic device attached into the insulin pen and connected with a smartphone app and has an insulin reminder system to reduce insulin omissions (137); this device helps to improve glycemic control and reduce glycemic variability with improved adherence in a recent pilot, randomized study among T1DM (138) and among T2DM patients (139). Another two devices do not connect with any mobile app but present an interactive display (Timesulin^®^) or flash diode (Dukada^®^ Trio), which define the time of last insulin injection (140, 141). The GoCap device received FDA approval (125). Clinical trials which compare different insulin pen caps are not available yet.

Cited studies related to smart insulin pens and their technical characteristics are summarized in **Tables 5** and **6**. As for the studies related to insulin pen caps and thier technical details the summery is provided in **Tables 7** and **8**, accordingly.

**Table 5:** Smart insulin pens.

Study, year	Device studied/device compared	Type of insulin/company (number of users)	Participants	Study design	Results
Adolfsson et al., 2020 (133)	NovoPen^®^ 6	Basal and/or bolus insulin: deguldec (n = 21), detemir (n = 1), aspart (n = 79), human insulin (n = 1), faster-acting insulin (n = 1)	94 participants (48 men and 46 women; aged 18–83 years, mean 40.1 years) with T1DM	Multicenter, prospective, observational, proof-of-concept study, Participants were using continuous glucose monitoring (CGM) and administered bolus and/or basal insulin with NovoPen^®^ 6. During each healthcare professional (HCP) visit, pen and CGM data were downloaded. The analysis included time in range (TIR; sensor glucose 3.9–10.0 mmol/l), time in hyperglycemia (>10 mmol/l), and hypoglycemia (L1: 3.0–<3.9 mmol/l; L2:<3.0 mmol/l). Missed bolus done (MBD) injections were meals without bolus injection within -15 and +60 min from the start of a meal. These outcomes were compared between the baseline (until visit 1) and follow-up periods (at least 5 HCP visits).	TIR increased (+1.9, 95% CI: 0.8–3.0 h/day, p < 0.001) from baseline to follow-up period with a reduction in time in hyperglycemia (-1.8; 95% CI:- 3.0–(-0.6) h/day, p = 0.003) and L2 hypoglycemia (-0.3; 95% CI: -0.6–(-0.1) h/day; p = 0.005) but with no change in time in L1 hypoglycemia. MBD injections decreased by 43% over the study (p = 0.002).
Jendle et al., 2021 (134)	NovoPen^®^ 6	Basal and/or bolus insulin: deguldec (n = 21), detemir (n = 1), aspart (n = 79), human insulin (n = 1), faster-acting insulin (n = 1)	94 participants (48 men and 46 women; aged 18–83 years, mean 40.1 years) with T1DM	Multicenter prospective, observational, proof-of-concept study, continuation of Swedish study (Adolfsson et al., 2020 (80)) Clinical outcomes and healthcare costs (in 2018 Swedish krona, SEK) were projected to estimate cost-effectiveness of smart insulin pen use over patients’ lifetime.	Smart insulin pen use was associated with improvement of mean discounted life expectancy (+0.90 years) and quality-adjusted life expectancy (+1.15 quality-adjusted life-years). Moreover, using smart injectors was a source of cost savings (direct SEK 124,270; indirect SEK 373,725) in comparison to standard care. The abovementioned profits were a result of projected lower frequency and delayed onset of diabetes complications versus standard care.
Vigersky et al., 2021 (142)	InPen™	Bolus insulin	529 individuals with non-optimal glycemic control (423 ones with glucose management indicator (GMI) >8.0% and 106 ones with GMI >9.5%)	Observational study CGM data were collected and compared before and up to 90 days after initiating InPen™ use. The outcomes were evaluated including means sensor glucose (SG), GMI, TIR, time above range (TAR), and time below range (TBR).	Patients with suboptimal metabolic control (GMI >8.0%) presented increased TIR (+2.3%, 0.6 h/day), reduced GMI (0.1%), SG (-4.3 mg/dl), and TAR (-2.4%) with no change in TBR, in comparison to pre-InPen™ use. Participants with poorest glycemic control at baseline (GMI >9.5) had TIR improvement by +5.0% (1.2 h/day), GMI by -0.4%, SG by -14.9 mg/dl, and TAR by 5.1% (1.2 h/day) with no change in TBR. From the first month to 90-days, post-InPen™ use bolus frequency decreased (from 3.7 to 3.6/day and 3.3 to 3.2/day, respectively) and total rapid-acting daily dose of insulin increased (from 26.29 to 27.19 U/day and 27.57 to 29.24 U/day, respectively). All mentioned results were significant (p < 0.05).

T1DM, type 1 diabetes mellitus; CGM, continuous glucose monitoring; HCP, healthcare professional; TIR, time in range (70–180 mg/dl; 3.9–10.0 mmol/l); GMI, glucose management indicator; SG, sensor glucose; TAR, time above range (>180 mg/dl; >10.0 mmol/l); TBR, time below range (<70 mg/dl; <3.9 mmol/l); U, units of insulin.

**Table 6:** Technical characteristics of smart insulin pens.

Pen device (year of introduction)	Company	Insulin producing company/insulin compatibilities		Cartridge volume and insulin concentration	Dose range (dose increment)	Monitors active insulin on board/bolus dose calculator	Reports to download/connects with company app on smartphones	Special characteristics	Battery lifetime/application service
InPen™ (2017) (123, 143)	Companion Medical	Lilly	Humalog^®^	3 ml (100 IU/ml)	0.5–30 units (0.5 unit)	Yes	Yes	Integrates with CGM, insulin injection reminder, temperature sensor	1 year/ Android, Apple
		Novo Nordisk	NovoLog^®^						
			Fiasp^®^						
ESYSTA^®^ BT pen (144)	Emperra	Novo Nordisk	NovoRapid^®^	3 ml (100 IU/ml)	1–60 units (1 unit)	Yes	Yes	Stores 1,000 records, displays of the last insulin dose	6 months, replaceable/ Android, Apple
			NovoMix^®^ 30						
			Levemir^®^						
			Actrapid^®^						
			Actraphane^®^ 30/-50						
			Protaphane^®^						
		Sanofi-Aventis	Lantus^®^						
			Apidra^®^						
			Insuman^®^ Rapid						
			Insuman^®^ Comb 15/-25/-50						
			Insuman^®^ Basal						
		Lilly	Huminsulin^®^ Normal						
			Huminsulin^®^ Profil III						
			Huminsulin^®^ Basal (NPH)						
			Humalog^®^						
			Humalog^®^ Mix 25/-50						
			Abasaglar^®^						
		Berlin-Chemie	Berlinsulin^®^ H Normal						
			Berlinsulin^®^ H 30/70						
			Berlinsulin^®^ H Basal						
			Liprolog^®^ Mix 25/-50 Pen						
			Liprolog^®^						
		B. Braun	Insulin B. Braun Rapid^®^						
			Insulin B. Braun Comb^®^						
			Insulin B. Braun Basal^®^						
Pendiq 2.0 (145)	Pendiq	Novo Nordisk	NovoRapid^®^	3 ml (100 IU/ml)	0.5–60 units (0.1 unit)	No	Yes	Low battery/insulin level alarms, data transmit to a computer with USB cable, stores 1,000 records	Rechargeable with USB charger/ Android, Apple
			Fiasp^®^						
			NovoLog^®^						
		Lilly	Humalog^®^						
		Sanofi-Aventis	Apidra^®^						
			Lispro^®^						
		Berlin-Chemie	Liprolog^®^						
NovoPen^®^ 6 (2019) (146)	Novo Nordisk	Novo Nordisk	NovoRapid^®^	3 ml (100 IU/ml)	1–60 units (1 unit)	Yes	Yes	Dose memory, uses NFC to transfer data	4 to 5 years/ Android, Apple
			NovoLog^®^						
			Actrapid^®^						
			Fiasp^®^						
			Levemir^®^						
			Tresiba^®^						

NFC, near-field communication.

**Table 7:** Insulin pen caps.

Study, year	Device studied/device compared	Type of insulin/company	Participants	Study design	Results
Gomez -Peralta F. et al. (2019) (137)	Insulclock^®^/none	Humulin NPH, Abasaglar, Humalog^®^, Humalog^®^ Junior, Humalog^®^ Mix25, Humalog^®^ Mix50, and Humalog^®^ 200/Eli Lilly	9 volunteers with T1DM	Performance and functionalities tests	Insulclock^®^ detected seven types of insulin pens with a 97% correct classification rate. Most of the doses were accurately detected (deviation = 0), with relative errors ranging from 3% to 7% across different dosages among 556 injections.
Gomez-Peralta F. et al. (2020) (138)	Insulclock^®^/standard pen (masked device)	Humalog^®^ KwikPen^®^/Eli Lilly	16	Randomized, single-center, prospective, open-label, pilot study	Insulclock^®^ led to the decrease in mean glucose (-27.0 mg/dl [1.5 mmol/l]; p = 0.013), glucose standard deviation (SD) (-14.4 mg/dl [0.8 mmol/l]; p = 0.003), and time above range (TAR) (-12.5%, p = 0.0026), and an increase in time in range (TIR) (+7%; p = 0.038) in the overall population.
Galindo et al. (2021) (139)	Insulclock^®^/standard pen (masked device)	Lantus®/Sanofi-Aventis	80 patients with uncontrolled T2DM on basal insulin	Randomized, 26-week, prospective, crossover, pilot study	Patients in the active phase were characterized by lower mean daily blood glucose (147.0 ± 34 vs. 157.6 ± 42 mg/dl, *p* < .01) and greater reduction of HbA1c (-0.98% vs. -0.72%, *p* = .006) but with no significant changes in treatment adherence, insulin omission, and insulin mistiming.

**Table 8:** Technical characteristics of insulin pen caps.

Smart insulin pen cap (year of introduction)	Company	Insulin producing company/insulin compatibilities			Mobile app/company	Bluetooth/USB	Special characteristics	Battery lifetime
Timesulin^®^ (2010) (141)	Bigfoot Biomedical	Eli Lilly	KwikPen^®^		No	Yes/no	Records the time since the last injection	1 year
		Novo Nordisk	FlexPen^®^					
			FlexTouch^®^					
		Sanofi-Aventis	SoloSTAR^®^					
Dukada^®^ Trio (2012) (140)	Dukada^®^	Novo Nordisk	FlexPen^®^		No	No/no	Flexible grip features, a light above the needle	6–8 months, replaceable
		Sanofi-Aventis	SoloSTAR^®^					
GoCap (2013) (136)	Common Sensing	Sanofi-Aventis	SoloSTAR^®^		Yes/Apple, Android	Yes/yes	Shows the quantity of insulin in the pen, time and type of insulin injection displays in app	10 -days, rechargeable with micro-USB cable
		Novo Nordisk	FlexPen^®^					
		Eli Lilly	KwikPen^®^					
Insulclock^®^ (2019) (137)	Insulcloud	Sanofi-Aventis	SoloSTAR^®^	Yes/Apple, Android		Yes/No	Indicates the time, type, and amount of insulin administrated. App remained about food/glucose input, temperature fluctuations	Rechargeable with micro-USB cable

## Conclusions

Insulin remains the primary medication in the treatment of T1DM and is often used therapy in T2DM. The methods and tools for insulin administration are various and have been constantly evolving for over the last 100 years. Insulin pens have changed the lives of millions of people who suffer from diabetes and now are the most widespread way of administering insulin. They are safe, simple to use, convenient, efficient, and less painful than conventional vials and syringes. An increasing number of modern, yet useful features may help to improve patients’ quality of life. Technology evolves to improve adherence and glycemic outcomes, optimize delivery, and reduce dosing errors. Studies performed up to date, summarized in this review, indicate that insulin pens came a long way from a very simple device produced in the year 1985 up till the newest insulin smart pens, and the further improvement is on the way.

## Author Contributions

Conceptualization: MMas, KN, and JG. Writing—original draft preparation: MMas, KN, OJ, HK, MMac, and JG. Review and editing: MMas, KN, OJ, HK, and JG. Visualization: MMas, OJ, and KN. All authors contributed to the article and approved the submitted version.

## Funding

There is no external funding for this review. Bioton S.A. company funded the publication fee only.

## Conflict of Interest

MMas works for Bioton S.A. KN and JG received lecture honoraria from Bioton S.A., Eli Lilly, Sanofi Aventis, Novo Nordisk and Polfa Tarchomin. HK received lecture honoraria from Sanofi Aventis.

The remaining authors declare that the research was conducted in the absence of any commercial or financial relationships that could be construed as a potential conflict of interest.

The reviewer MH declared a shared affiliation with the authors to the handling editor at the time of review.

## Publisher’s Note

All claims expressed in this article are solely those of the authors and do not necessarily represent those of their affiliated organizations, or those of the publisher, the editors and the reviewers. Any product that may be evaluated in this article, or claim that may be made by its manufacturer, is not guaranteed or endorsed by the publisher.
